# Immunogenicity and safety of 4 vs. 3 standard doses of HBV vaccination in HIV-infected adults with isolated anti-HBc antibody

**DOI:** 10.1186/s12981-019-0225-3

**Published:** 2019-05-03

**Authors:** Nattinee Laksananun, Jutarat Praparattanapan, Wilai Kotarathititum, Khuanchai Supparatpinyo, Romanee Chaiwarith

**Affiliations:** 1 Division of Infectious Diseases, Department of Medicine, Faculty of Medicine, Chiang Mai University, Chiang Mai, 50200 Thailand; 2 Research Institute for Health Science, Chiang Mai University, Chiang Mai, Thailand

**Keywords:** HIV-infected adults, Isolated anti-HBc antibody, HBV vaccination, Immunological response, Anamnestic response

## Abstract

**Background:**

Presence of isolated anti-HBc antibody is common in HIV-infected patients in endemic areas and could be caused by prior HBV infection with loss of anti-HBs antibody. The role of vaccination in these patients remains controversial and is based largely on limited and low quality data. We, therefore, conducted this study to determine immunogenicity and safety of 4 vs. 3 standard doses of HBV vaccination in HIV-infected adults with isolated anti-HBc antibody.

**Methods:**

An open-label, randomized controlled trial was conducted among HIV-infected patients visiting HIV clinic of the Faculty of Medicine, Chiang Mai University, Chiang Mai, Thailand between July and September 2017. Inclusion criteria included ≥ 18 years of age, currently on a stable antiretroviral regimen, CD4+ cell count ≥ 200 cells/mm^3^, plasma HIV-1 RNA < 20 copies/mL, and isolated anti-HBc antibody. The participants were randomized to receive either 3 standard doses (20 µg at month 0, 1, 6) or 4 standard-doses (20 µg at month 0, 1, 2, 6) of IM HBV vaccination, and were evaluated for anamnestic response at week 4 and vaccine response at week 28.

**Results:**

Of the 97 patients screened, 54 (32 male, mean age of 46 years) were enrolled and 27 were allocated to each of the vaccination groups. Anamnestic response occurred in 25.9% vs. 33.3% in 3-dose group vs. 4-dose group, respectively (p = 0.551). The vaccine response rates at week 28 were 85.2% in 3-dose group vs. 88.9% in 4-dose group (p = 1.000); geometric mean titer of anti-HBs antibody at week 28 was 63.8 and 209.8 mIU/mL in 3-dose group and 4-dose group, respectively (p = 0.030). No adverse events were reported.

**Conclusions:**

An anamnestic response occurred in one-third of Thai HIV-infected patients with isolated anti-HBc antibody who received one dose of HBV vaccination; however, the majority were still unprotected. The use of either 3 or 4 standard-doses of vaccination was highly effective and should be recommended in all HIV-infected individuals with isolated anti-HBc antibody.

*Trial registration* ClinicalTrials.gov; NCT03212911. Registered 11 July 2019, https://clinicaltrials.gov/ct2/show/NCT03212911

**Electronic supplementary material:**

The online version of this article (10.1186/s12981-019-0225-3) contains supplementary material, which is available to authorized users.

## Background

Hepatitis B virus infection is common in HIV-infected patients, particularly in endemic areas for both viruses. HIV and HBV co-infection also causes higher morbidity and mortality than each infection alone, resulting in high rates of HBV reactivation and replication, acceleration of HBV progression to chronic hepatitis, cirrhosis and eventually hepatocellular carcinoma [1–4]. Therefore, HBV vaccination is strongly recommended in all HIV-infected patients with no evidence of prior HBV exposure or immunity [3, 4]. However, HIV-infected patients were found to have decreased serological response to HBV vaccination comparing to normal individuals (18–85% vs. > 90%) [5–9], with faster rates of antibody decline after acquiring protective anti-HBs Ab titers [3, 10, 11]. Various studies have been conducted to determine the optimal HBV vaccination regimen that induced the best immunologic response. The newly proposed regimens: 3 double doses (40 mcg at month 0, 1, 6), 4 double doses (40 mcg at month 0, 1, 2, 6) and 4 standard doses (20 mcg at month 0, 1, 2, 6), resulted in higher response rates [12–14]. In contrast, a study in Thailand [15] found that the standard vaccination schedule was highly effective (with a response rate of 88.6%). The 4-double-dose and 4-standard-dose regimens could not significantly increase the response rate (95.4% and 93.2%, respectively).

Presence of isolated anti-HBc antibody is common in HIV-infected patients and could be caused by prior HBV infection with loss of anti-HBs antibody or an occult HBV infection with low level and intermittent viremia [9, 16–23]. Those in the prior group may be at risk of reactivation or reinfection due to the lack of protective immunity and strongly require HBV vaccination [24–26]. Anamnestic response to a single dose of HBV vaccination in these population was low (7–32.5%) [9, 27–29] compared to those without HIV (41.6%) [27, 30]. Hence, a single-dose HBV vaccination is not recommended. Although the European and the DHHS guidelines currently recommend giving a 3 standard doses of HBV vaccination in HIV-infected patients with isolated anti-HBc antibody without routine checking of HBV DNA [31, 32], these are based on limited and low quality data. Three trials conducted in the US [9], Switzerland [28] and Italy [33] demonstrated 63%, 60% and 52.6% response rates to 3 standard doses of HBV vaccination, respectively, while a trial in France [34] showed an 89% response rate to a reinforced 3-double-dose vaccination after showing no response to a single standard dose. However, no randomized controlled trial or head-to-head comparison trial was available, and a trial on a 4-standard-dose regimen has never been conducted in this population.

Therefore, we conducted this randomized controlled trial to compare the immunogenicity at week 28 and safety of 4 vs. 3 standard doses of HBV vaccination in HIV-infected patients with isolated anti-HBc antibody. Secondary objectives were to determine the percentage of participants with anamnestic response at week 4, the percentage of high-level responders (with anti-HBs Ab ≥ 100 mIU/mL) at week 28, the intensity and frequency of vaccine adverse event (AE), the geometric mean titers of anti-HBs antibody at week 28; and the factors associated with anamnestic response at week 4 and vaccine response at week 28 of both vaccination regimens.

## Methods

### Study design, population and randomization

An open-label randomized controlled trial was conducted by recruiting HIV-infected patients visiting the HIV clinic of the Maharaj Nakorn Chiang Mai Hospital, Faculty of Medicine, Chiang Mai University, Chiang Mai, Thailand, between July and September 2017. Inclusion criteria were (1) HIV-infected individual, (2) ≥ 18 years old, (3) currently taking combination antiretroviral therapy (cART), (4) CD4+ cell count ≥ 200 cell/mm^3^, (5) plasma HIV RNA < 20 copies/mL for at least 1 year, (6) isolated anti-HBc antibody (negative for HBs antigen and anti-HBs antibody), (7) negative for anti-HCV antibody, and (8) provided written informed consent. Exclusion criteria were (1) pregnant or breast-feeding women, (2) intolerance to any component of HBV vaccine, (3) history of HBV vaccination, (4) liver enzymes ≥ 5 upper normal limits in the past 3 months, (5) active AIDS-defining opportunistic infection, (6) active malignancy with current chemotherapy or radiotherapy, (7) systemic steroid therapy (≥ 0.5 mg/kg/day) or any immunomodulating therapy in the last 6 months, (8) other immunocompromised disorders (e.g. solid organ transplant), (9) asplenism, (10) renal insufficiency (CrCl ≤ 30 mL/min), and (11) decompensated cirrhosis (Child–Pugh C).

Eligible participants were randomized, with 1:1 allocation ratio and a block size of 4, into 2 groups: (1) 3-dose group receiving recombinant HBV vaccine, produced by the Center for Genetic Engineering and Biotechnology, Cuba (Heberbiovac-HB) 20 µg IM at month 0, 1 and 6; or (2) 4-dose group receiving the same HBV vaccine 20 µg IM at month 0, 1, 2, and 6. Each participant received a diary to record the intensity and frequency of vaccine-related adverse events, containing checklists of occurrence and severity of local reactions at injection sites (edema, redness, pain) or systemic reactions (fever, fatigue, headache) up to 7 days after vaccination. Baseline data were collected including sex, age, contraception method, risk of HIV acquisition, history of intravenous drug use (IVDU) or men who have sex with men (MSM) status, time since HIV diagnosis, cART regimen(s), duration of cART, and all previous results of CD4+ cell counts and plasma HIV-1 RNA. Following laboratory tests were performed at baseline: hepatitis markers (anti-HBs antibody, HBs antigen, anti-HBc antibody, and anti-HCV Ab using CMIA technique by ARCHITECT *i*2000SR immunoassay analyzer (Abbott Ireland Diagnostics Division, Sligo, Ireland), CD4+ cell count using BD Tritest three-color reagents, and plasma HIV-a RNA using Roche Cobas AmpliPrep/Cobas Taqman HIV-1 Test, version 2.0, with a limit of detection of 20 copies/mL. At weeks 4 and 28 after the first dose of vaccine, plasma was tested for anti-HBs Ab titer. All tests were performed by technical staff masked to the participants’ group allocation.

The study was approved by the Research Ethics Committee of Faculty of Medicine Chiang Mai University. This trial was registered on ClinicalTrials.gov; NCT03212911 on July 11, 2017. (URL: https://clinicaltrials.gov/ct2/show/NCT03212911).

### Definitions of terms

*Anamnestic response* is defined as having anti-HBs antibody ≥ 10 mIU/mL at week 4 after the first dose of vaccine [9, 33, 34].

*Vaccine response* is defined as having anti-HBs ≥ 10 mIU/mL at week 28 [9, 15, 33, 34].

*High*-*level response* is defined as anti-HBs antibody ≥ 100 mIU/mL at week 28 [15, 34].

*cART* active against HBV is defined as a regimen containing either lamivudine, emtricitabine, or tenofovir [35].

*Occult HBV infection* is the presence of HBV DNA in serum without HBs antigen [33, 36].

### Statistical analysis

Categorical data were presented as frequency and percentage (%), and continuous data as mean ± standard deviation (SD) or median (interquartile range: IQR) as appropriate. Chi-square test or Fisher’s exact test were used to compare proportions between groups, while Student’s T-test or Mann–Whitney U test were used to compare continuous data. Predictive factors associated with response to HBV vaccination were tested in logistic regression analysis and characteristics with p < 0.10 in the univariate analysis were included in multivariate models on the basis of a backward-stepwise procedure. A 2-sided test was used to indicate statistical significance at p-value of < 0.05. Analyses were based on the intention to treat. All statistical analyses were performed using StataCorp. 2015. Stata Statistical Software: Release 14. College Station, Tx: StataCorp LP.

From previous findings, we estimated that the percentage of responders in the 3-standard-dose vaccination would be 60% [9, 28], compared to 90% in the 4-standard-dose vaccination at week 28 [34]. In order to detect the difference with 90% power and 2-sided α of 0.05, a sample size of 48 participants per group was required, with a total number of 96 participants.

## Results

**Fig. 1:**
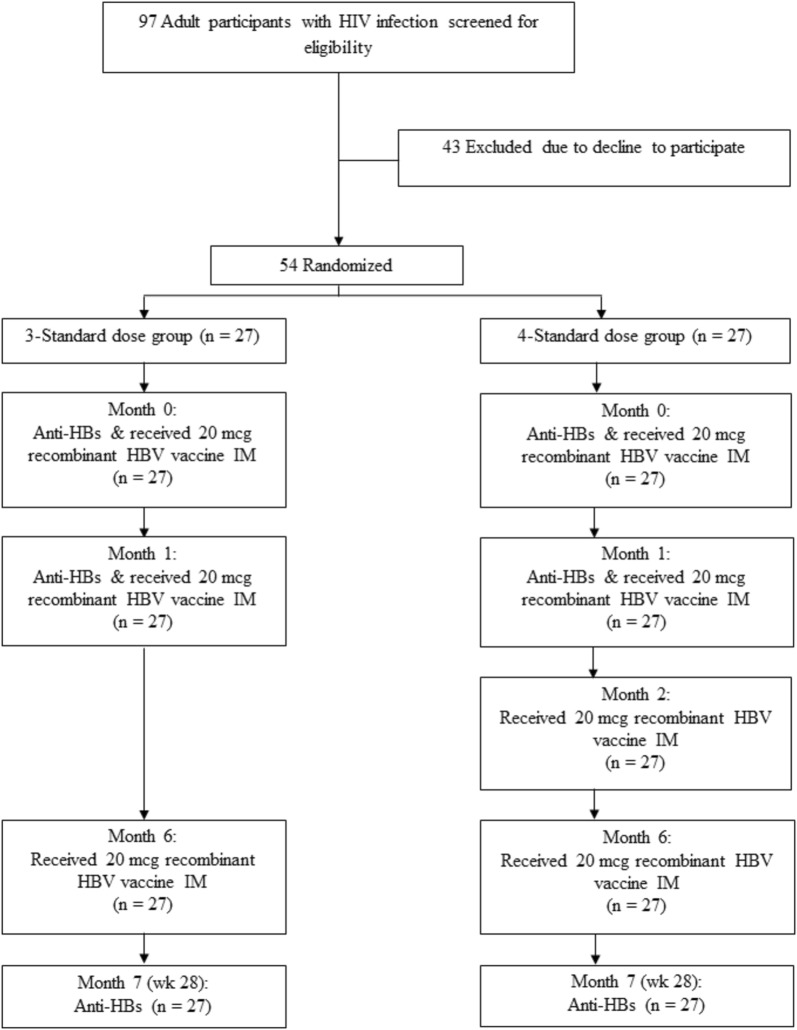
Consort diagram of study participants

From July to September 2017, 97 HIV-infected patients were screened for eligibility; 43 patients declined to participate the study. The remaining 54 participants were enrolled and 27 each were randomized to receive 3 or 4 standard-doses of HBV vaccination (Fig. 1). There were no dropouts throughout the trial.

### Baseline characteristics

Of the 54 enrolled participants, 32 were male (59.3%). The mean age in the 3-dose and 4-dose groups was 45.8 ± 13.5 years and 46.6 ± 11.0 years, respectively. Baseline characteristics of participants in both vaccination groups are shown in Table 1. There was no statistically significant difference in any factor between the 2 groups.

**Table 1:** Baseline characteristics of study participants

Baseline characteristics	3-dose group (n = 27)	4-dose group (n = 27)	p-value
Age, years (mean ± SD)	45.8 ± 13.5	46.6 ± 11.0	0.826
Male	16 (59.3%)	16 (59.3%)	1.000
Risk of HIV acquisition			0.487
Heterosexual	19 (70.4%)	21 (77.8%)	
Homosexual (MSM)	7 (25.9%)	5 (18.5%)	
Blood transfusion	1 (3.7%)	0	
IVDU	0	1 (3.7%)	
Time since HIV diagnosis, years (mean ± SD)	12.4 ± 6.2	11.3 ± 6.4	0.534
Duration of cART, years (mean ± SD)	10.0 ± 4.8	9.4 ± 4.7	0.691
CD4+ cell count, cells/mm^3^ (median (IQR)	611 (429, 853)	534 (392, 829)	0.697
Nadir CD4+ cell count, cells/mm^3^ (median (IQR)	148 (52, 323)	110 (52, 228)	0.392
Nadir CD4+ cell count < 200 cells/mm^3^	19 (70.4%)	18 (66.7%)	0.770
Current cART active against HBV	27 (100%)	27 (100%)	
cART regimen			1.000
NNRTI-based	25 (92.6%)	25 (92.6%)	
PI-based	2 (7.4%)	2 (7.4%)	
NRTI/NtRTI backbone			1.000
TDF/FTC	24 (88.9%)	23 (85.2%)	
TDF + 3TC	1 (3.7%)	1 (3.7%)	
AZT/3TC	1 (3.7%)	2 (7.4%)	
ABC/3TC	1 (3.7%)	1 (3.7%)	

*3TC* lamivudine, *ABC* abacavir, *AZT* zidovudine, *cART* combination antiretroviral therapy, *FTC* emtricitabine, *IQR* interquartile range, *IVDU* intravenous drug use, *MSM* men who have sex with men, *NRTI* nucleoside reverse transcriptase inhibitor, *NNRTI* non-nucleoside reverse transcriptase inhibitor, *NtRTI* nucleotide reverse transcriptase inhibitor, *PI* protease inhibitor, *SD* standard deviation, *TDF* tenofovir disoproxil fumarate

### Anamnestic response to HBV vaccination

At week 4 after the first dose of vaccination, anamnestic response occurred in 25.9% (95% CI 11.1–46.3) in 3-dose group vs. 33.3% (95% CI 16.5–54.0) in 4-dose group (p = 0.551). There was only 1 participant with a high-level response in the 4-dose group (3.7%) (Table 2). The geometric mean titer (GMT) of anti-HBs antibody at week 4 in the 3-dose group was 4.4 mIU/mL compared to 5.3 mIU/mL in the other group (p = 0.714).

**Table 2:** Hepatitis B vaccination response

Outcomes	3-dose group (n = 27)	4-dose group (n = 27)	p-value
Anamnestic response	7 (25.9%)	9 (33.3%)	0.551
High-level responders at week 4	0 (0%)	1 (3.7%)	1.000
Responders at week 28	23 (85.2%)	24 (88.9%)	1.000
High-level responders at week 28	12 (44.4%)	17 (63.0%)	0.172
GMT of anti-HBs Ab at week 4 (95% CI)	4.4 (2.1–8.4)	5.29 (2.4–10.8)	0.714
GMT of anti-HBs Ab at week 28 (95% CI)	63.8 (31.4–128.7)	209.8 (90.0–487.4)	0.030

*CI* confidence interval, *GMT* geometric mean titer

Compared with those who did not have anamnestic response, those who had anamnestic response were younger (age 38.6 ± 11.6 years compared to 49.4 ± 11.2 years; p = 0.002), had shorter time since HIV diagnosis (8.5 years vs. 14.0 years; p = 0.006), shorter duration of cART (7 years vs. 12 years; p = 0.002) and higher nadir CD4+ cell count (198 cells/mm^3^ vs. 103 cells/mm^3^; p = 0.029). The vaccination regimen, sex, risk of HIV acquisition, current CD4 cell count, or cART regimen were not found to be different (Additional file 1: Table S1). In multivariate analysis, only age < 45 years old and a nadir CD4+ cell count ≥ 100 cells/mm^3^ were independently predictive of anamnestic response with an odd ratio (OR) of 17.4 (95% CI 3.0–102.0, p = 0.002) and 21.6 (95% CI 2.7–170.4, p = 0.004), respectively.

### Vaccine response to HBV vaccination

**Fig. 2:**
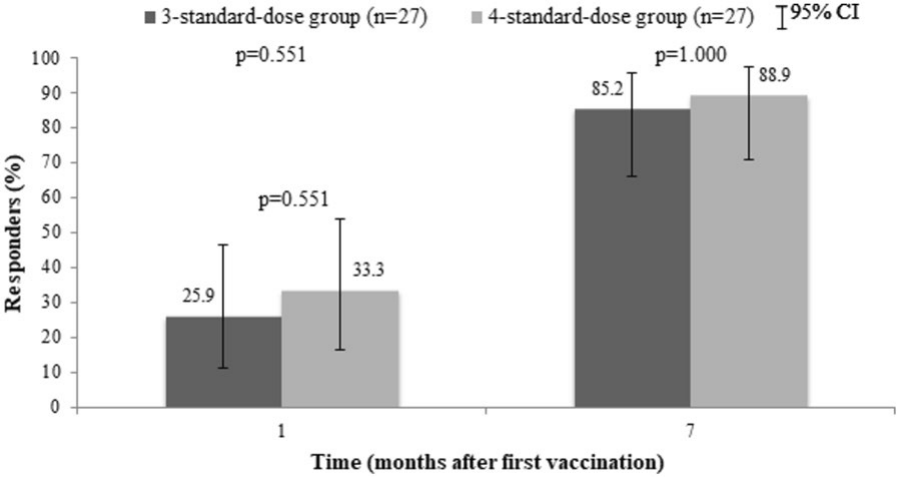
Percentages of responders (anti-HBs ≥ 10 mIU/mL) by vaccination regimen

**Fig. 3:**
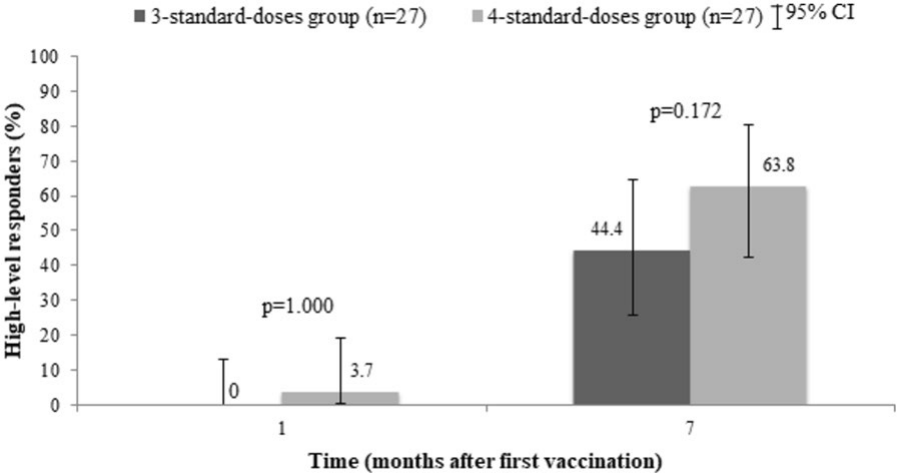
Percentages of high-level responders (anti-HBs ≥ 100 mIU/mL) by vaccination regimen

**Fig. 4:**
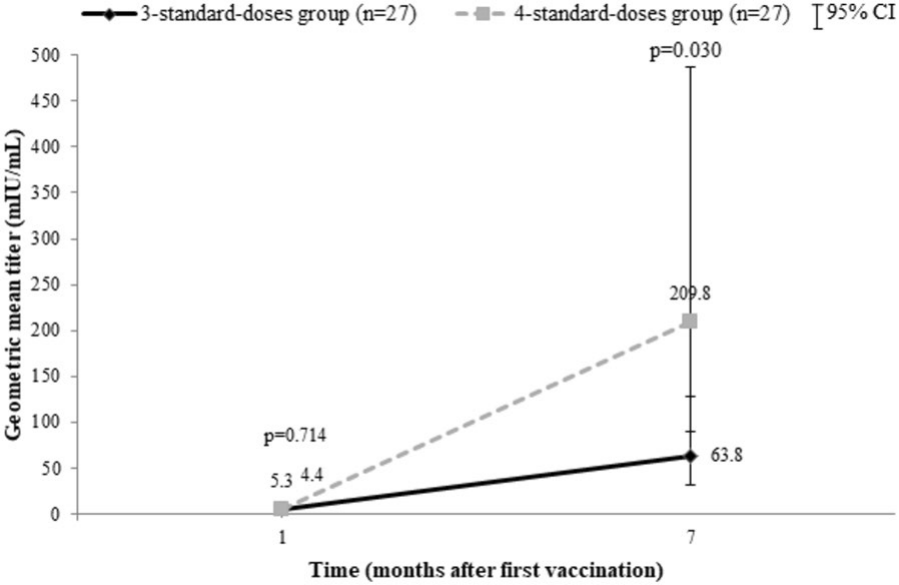
Geometric mean titer (GMT) of anti-HBs by vaccination regimen

At week 28 after the first dose of vaccination, 85.2% (95% CI 66.3–95.8) and 88.9% (95% CI 70.8–97.6) of participants in the 3-dose and 4-dose groups had anti-HBs ≥ 10 mIU/mL, respectively (p = 1.000), as shown in Fig. 2. High-level response occurred in 44.4% (95% CI 25.5–64.7) of participants in the 3-dose group compared to 63.0% (95% CI 42.4–80.6) in the 4-dose group (p = 0.172) (Fig. 3). The geometric mean titer (GMT) of anti-HBs antibody at week 28 was statistically significant between the 2 groups (63.8 mIU/mL and 209.8 mIU/mL in 3-dose and 4-dose groups, respectively, p = 0.030) (Fig. 4).

Of all factors evaluated, none were found to be predictive of response to vaccination at week 28, with statistical significance (Additional file 1: Table S2).

### Adverse events

No local (edema, redness, and pain) or systemic (fever, fatigue, headache) adverse events to either vaccination regimen were reported in this trial.

## Discussion

In this study of HBV vaccination regimens in Thai HIV-infected patients with isolated anti-HBc antibody, the response rate at week 28 after the first dose of vaccination in the 3-standard-dose regimen was very high (85.2%), and the response rate to the 4-standard-dose vaccination schedule was similar (88.9%). This finding differed from previous trials done in Italy, the US, and Switzerland, of which the vaccine response rates were quite low, varying from 52.6 to 63% [9, 28, 33]. Nonetheless, this high vaccine response was similar to the finding in another trial conducted in Thai HIV-infected patients without any HBV markers; that study demonstrated 88.6% response to the 3-standard-dose, 93.2% to the 4-standard-dose and 95.4% to the 4-double-dose regimen [15]. The vaccine response rate to the standard HBV vaccination from both Thai studies were almost as high as that from most studies in non-HIV individuals (> 90%) [9, 11, 37, 38]. It is possible that certain genetic or environment factors may play a role in variation of vaccination response between the Asian and Caucasian population. Our study also demonstrated that the geometric mean titer at week 28 after the first dose of vaccination was significantly higher in the 4-standard-dose than that in the 3-standard-dose group. This finding was similar to previous studies and might reflect a more durable immunological response after adding another dose of vaccine to or doubling the dose of vaccine in the vaccination regimen; however, a confirmation by longer follow-up of anti-HBs antibody titer is needed to evaluate for long-term persistence of immunity. This study could not demonstrate any predictive factors for favorable vaccine response at week 28 after the first dose of vaccination; this is similar to another study [28].

For the anamnestic response to a single-dose HBV vaccination in HIV-infected patients with isolated HBc antibody, the rates of 25.9% and 33.3% among the 3-standard-dose and 4-standard-dose groups, respectively, in our study were higher than the rate of 7% in a previous trial conducted in the metropolitan area of Thailand (7%) [29], but were corresponded with those in other studies from various regions [9, 27, 28, 33]. One trial has demonstrated that HIV acquisition through IVDU was a predictive factor for favorable anamnestic response [28]. However, in that trial the proportion of IVDU population was much higher than in our study (43% compared to 1.9%). On the other hand, our study found that age < 45 years old was a predictive factor of higher anamnestic response. This could possibly be explained by stronger immunological memory in younger individuals [39–41]. A nadir CD4+ cell count ≥ 100 cells/mm^3^ was also found to be a predictor of higher anamnestic response. This finding is logical since better immunological response could be expected in those with higher nadir CD4+ cell count [42, 43]. Although the anamnestic response occurred considerably in both of the study groups in this study, the majority of patients still remained unprotected after a single-dose HBV vaccination. Therefore, a single dose vaccination is not adequate and could not be recommended as a standard practice.

This study confirms that HBV vaccination, either using 3 standard doses or adding another dose of vaccine, is considerably safe. There were no serious local or systemic adverse events reported.

There are several limitations of this study. First, the sample size recruited to the study is smaller than the recruitment plan, and this could affect the power of the trial to detect some differences which may exist. However, this sample size limitation occurs similarly in most trials conducted to evaluate this population. A multi-center trial should be done to overcome this limitation. Second, the HBV DNA test was not performed. However, 90.7% of the study population received tenofovir disoproxil fumarate (TDF) and emtricitabine (FTC) or lamivudine (3TC). Not performing HBV DNA test is unlikely to affect the outcomes. Third, we were unable to perform longer follow-up for anti-HBs antibody titers to evaluate immune persistence; a plan to follow up at 1 year or longer after vaccination is underway.

## Conclusions

This study demonstrated that in Thai HIV-infected patients with isolated anti-HBc antibody, anamnestic response occurred considerably in both vaccination regimens, but the majority of population still remained unprotected. Hence, a single dose vaccination is not recommended. The usual 3 standard doses of HBV vaccination was highly effective with a high response rate, and adding another standard dose to the vaccination regimen can slightly improve this rate without any statistical significance. However, the 4-dose regimen produces significantly higher antibody titer that may have benefit in the long run. The use of either 3 or 4 standard-doses of HBV vaccination was highly effective and safe; this should be recommended in all HIV-infected individuals with isolated anti-HBc antibody.

## Additional file

**Additional file 1: Table S1.** Comparison of characteristics between those who had and did not have anamnestic response (anti-HBs ≥ 10 mIU/mL at week 4). **Table S2.** Comparison of characteristics between responders and non-responders (anti-HBs ≥ 10 mIU/mL) at week 28.

## Abbreviations

Ab: antibody
ABC: abacavir
anti-HBc: anti-hepatitis B core
anti-HBs: anti-hepatitis B surface
AZT: zidovudine
cART: combination antiretroviral therapy
CMIA: chemiluminescent magnetic microparticle immunoassay
CrCl: creatinine clearance
DNA: deoxyribonucleic acid
FTC: emtricitabine
GMT: geometric mean titer
HBs: hepatitis B surface
HBV: hepatitis B virus
HIV: human immunodeficiency virus
IM: intramuscular
IQR: interquartile range
IVDU: intravenous drug use
mcg: microgram
mIU/mL: milli-international units per milliliter
mm^3^: cubic millimeter
NRTI: nucleoside reverse transcriptase inhibitor
NNRTI: non-nucleoside reverse transcriptase inhibitor
NtRTI: nucleotide reverse transcriptase inhibitor
OR: odds ratio
PI: protease inhibitor
SD: standard deviation
TDF: tenofovir disoproxil fumarate

## References

[1] Salmon-Ceron D, Lewden C, Morlat P, Bevilacqua S, Jougla E, Bonnet F (2005). Liver disease as a major cause of death among HIV infected patients: role of hepatitis C and B viruses and alcohol. J Hepatol.

[2] Thio CL, Seaberg EC, Skolasky R, Phair J, Visscher B, Munoz A (2002). HIV-1, hepatitis B virus, and risk of liver-related mortality in the multicenter cohort study (MACS). Lancet.

[3] Kim HN, Harrington RD, Crane HM, Dhanireddy S, Dellit TH, Spach DH (2009). Hepatitis B vaccination in HIV-infected adults: current evidence, recommendations and practical considerations. Int J STD AIDS.

[4] Kaplan JE, Benson C, Holmes KK, Brooks JT, Pau A, Masur H (2009). Guidelines for prevention and treatment of opportunistic infections in HIV-infected adults and adolescents: recommendations from CDC, the National Institutes of Health, and the HIV Medicine Association of the Infectious Diseases Society of America. MMWR Recomm Rep.

[5] Fuster F, Vargas JI, Jensen D, Sarmiento V, Acuna P, Peirano F (2016). CD4/CD8 ratio as a predictor of the response to HBV vaccination in HIV-positive patients: a prospective cohort study. Vaccine.

[6] Landrum ML, Huppler Hullsiek K, Ganesan A, Weintrob AC, Crum-Cianflone NF, Barthel RV (2009). Hepatitis B vaccine responses in a large U.S. military cohort of HIV-infected individuals: another benefit of HAART in those with preserved CD4 count. Vaccine.

[7] Pettit NN, De Pestel DD, Malani PN, Riddell JT (2010). Factors associated with seroconversion after standard dose hepatitis B vaccination and high-dose revaccination among HIV-infected patients. HIV Clin Trials.

[8] Potsch DV, Camacho LA, Tuboi S, Villar LM, Miguel JC, Ginuino C (2012). Vaccination against hepatitis B with 4-double doses increases response rates and antibodies titers in HIV-infected adults. Vaccine.

[9] Gandhi RT, Wurcel A, Lee H, McGovern B, Shopis J, Geary M (2005). Response to hepatitis B vaccine in HIV-1-positive subjects who test positive for isolated antibody to hepatitis B core antigen: implications for hepatitis B vaccine strategies. J Infect Dis.

[10] Pasricha N, Datta U, Chawla Y, Singh S, Arora SK, Sud A (2006). Immune responses in patients with HIV infection after vaccination with recombinant Hepatitis B virus vaccine. BMC Infect Dis.

[11] Collier AC, Corey L, Murphy VL, Handsfield HH (1988). Antibody to human immunodeficiency virus (HIV) and suboptimal response to hepatitis B vaccination. Ann Intern Med.

[12] Fonseca MO, Pang LW, de Paula Cavalheiro N, Barone AA, Heloisa Lopes M (2005). Randomized trial of recombinant hepatitis B vaccine in HIV-infected adult patients comparing a standard dose to a double dose. Vaccine.

[13] Launay O, van der Vliet D, Rosenberg AR, Michel ML, Piroth L, Rey D (2011). Safety and immunogenicity of 4 intramuscular double doses and 4 intradermal low doses vs standard hepatitis B vaccine regimen in adults with HIV-1: a randomized controlled trial. JAMA.

[14] Rey D, Krantz V, Partisani M, Schmitt MP, Meyer P, Libbrecht E (2000). Increasing the number of hepatitis B vaccine injections augments anti-HBs response rate in HIV-infected patients. Effects on HIV-1 viral load. Vaccine.

[15] Chaiklang K, Wipasa J, Chaiwarith R, Praparattanapan J, Supparatpinyo K (2013). Comparison of immunogenicity and safety of four doses and four double doses vs. standard doses of hepatitis B vaccination in HIV-infected adults: a randomized, controlled trial. PLoS ONE.

[16] Filippini P, Coppola N, Pisapia R, Scolastico C, Marrocco C, Zaccariello A (2006). Impact of occult hepatitis B virus infection in HIV patients naive for antiretroviral therapy. AIDS.

[17] Shire NJ, Rouster SD, Rajicic N, Sherman KE (2004). Occult hepatitis B in HIV-infected patients. J Acquir Immune Defic Syndr.

[18] Tsui JI, French AL, Seaberg EC, Augenbraun M, Nowicki M, Peters M (2007). Prevalence and long-term effects of occult hepatitis B virus infection in HIV-infected women. Clin Infect Dis.

[19] Azadmanesh K, Mohraz M, Aghakhani A, Edalat R, Jam S, Eslamifar A (2008). Occult hepatitis B virus infection in HIV-infected patients with isolated hepatitis B core antibody. Intervirology.

[20] Karaosmanoglu HK, Aydin OA, Nazlican O (2013). Isolated anti-HBc among HIV-infected patients in Istanbul, Turkey. HIV Clin Trials.

[21] Neau D, Winnock M, Jouvencel AC, Faure M, Castera L, Legrand E (2005). Occult hepatitis B virus infection in HIV-infected patients with isolated antibodies to hepatitis B core antigen: Aquitaine cohort, 2002–2003. Clin Infect Dis.

[22] Santos EA, Yoshida CF, Rolla VC, Mendes JM, Vieira IF, Arabe J (2003). Frequent occult hepatitis B virus infection in patients infected with human immunodeficiency virus type 1. Eur J Clin Microbiol Infect Dis.

[23] Witt MD, Lewis RJ, Rieg G, Seaberg EC, Rinaldo CR, Thio CL (2013). Predictors of the isolated hepatitis B core antibody pattern in HIV-infected and -uninfected men in the multicenter AIDS cohort study. Clin Infect Dis.

[24] Waite J, Gilson RJ, Weller IV, Lacey CJ, Hambling MH, Hawkins A (1988). Hepatitis B virus reactivation or reinfection associated with HIV-1 infection. AIDS.

[25] Pei R, Grund S, Verheyen J, Esser S, Chen X, Lu M (2014). Spontaneous reactivation of hepatitis B virus replication in an HIV coinfected patient with isolated anti-Hepatitis B core antibodies. Virol J.

[26] Bani-Sadr F, Maillard A, Ponscarme D, Scieux C, Molina JM (2003). Reactivation of HBV replication in HIV-HBV infected patients. Am J Med.

[27] Chakvetadze C, Bani-Sadr F, Le Pendeven C, Lescure FX, Fontaine C, Galperine T (2010). Serologic response to hepatitis B vaccination in HIV-Infected patients with isolated positivity for antibodies to hepatitis B core antigen. Clin Infect Dis.

[28] Kaech C, Pache I, Burgisser P, Elzi L, Darling KE, Cavassini M (2012). Immune response to hepatitis B vaccination in HIV-positive adults with isolated antibodies to HBV core antigen. J Infect.

[29] Jongjirawisan Y, Ungulkraiwit P, Sungkanuparph S (2006). Isolated antibody to hepatitis B core antigen in HIV-1 infected patients and a pilot study of vaccination to determine the anamnestic response. J Med Assoc Thai.

[30] Ural O, Findik D (2001). The response of isolated anti-HBc positive subjects to recombinant hepatitis B vaccine. J Infect.

[31] Rockstroh JK, Bhagani S, Benhamou Y, Bruno R, Mauss S, Peters L (2008). European AIDS Clinical Society (EACS) guidelines for the clinical management and treatment of chronic hepatitis B and C coinfection in HIV-infected adults. HIV Med.

[32] Guidelines for the prevention and treatment of opportunistic infections in HIV-infected adults and adolescents: recommendations from the Centers for Disease Control and Prevention, the National Institutes of Health, and the HIV Medicine Association of the Infectious Diseases Society of America. http://aidsinfo.nih.gov/contentfiles/lvguidelines/adult_oi.pdf. Accessed 7 Aug 2017.

[33] Morsica G, Bagaglio S, Spagnuolo V, Castagna A, Di Serio C, Galli A (2017). Immune response to hepatitis B vaccination in HIV-positive individuals with isolated antibodies against hepatitis B core antigen: results of a prospective Italian study. PLoS ONE.

[34] Piroth L, Launay O, Michel ML, Bourredjem A, Miailhes P, Ajana F (2016). Vaccination against hepatitis B virus (HBV) in HIV-1-infected patients with isolated anti-HBV core antibody: the ANRS HB EP03 CISOVAC prospective study. J Infect Dis.

[35] Guidelines for the Use of Antiretroviral Agents in Adults and Adolescents Living with HIV. Department of Health and Human Services. 2017. http://aidsinfo.nih.gov/contentfiles/lvguidelines/AdultandAdolescentGL.pdf. Accessed 30 Mar 2018.

[36] Raimondo G, Pollicino T, Cacciola I, Squadrito G (2007). Occult hepatitis B virus infection. J Hepatol.

[37] Carne C, Weller I, Waite J, Briggs M, Pearce F, Adler M (1987). Impaired responsiveness of homosexual men with HIV antibodies to plasma derived hepatitis B vaccine. Br Med J (Clin Res Ed).

[38] Wong EK, Bodsworth NJ, Slade MA, Mulhall BP, Donovan B (1996). Response to hepatitis B vaccination in a primary care setting: influence of HIV infection, CD4+ lymphocyte count and vaccination schedule. Int J STD AIDS.

[39] Fisman DN, Agrawal D, Leder K (2002). Effect of age on immunologic response to recombinant hepatitis B vaccine: a meta-analysis. Clin Infect Dis.

[40] Hamborsky J, Kroger A, Wolfe S, Control CFD, Prevention (2015). Epidemiology and prevention of vaccine-preventable diseases.

[41] Yang S, Tian G, Cui Y, Ding C, Deng M, Yu C (2016). Factors influencing immunologic response to hepatitis B vaccine in adults. Sci Rep.

[42] McKinnon LR, Kimani M, Wachihi C, Nagelkerke NJ, Muriuki FK, Kariri A (2010). Effect of baseline HIV disease parameters on CD4+ T cell recovery after antiretroviral therapy initiation in Kenyan women. PLoS ONE.

[43] Negredo E, Massanella M, Puig J, Pérez-Alvarez N, Gallego-Escuredo JM, Villarroya J (2010). Nadir CD4 T cell count as predictor and high CD4 T cell intrinsic apoptosis as final mechanism of poor CD4 T cell recovery in virologically suppressed HIV-infected patients: clinical implications. Clin Infect Dis.

