# Supplementary material for: Immunogenicity and safety of 4 vs. 3 standard doses of HBV vaccination in HIV-infected adults with isolated anti-HBc antibody
Source: AIDS Res Ther. 2019 May 3;16:10. doi: 10.1186/s12981-019-0225-3 (PMC6498566; doi:10.1186/s12981-019-0225-3)
Supplement: Supplementary file 1 — Additional file 1: Table S1. Comparison of characteristics between those who had and did not have anamnestic response (anti-HBs ≥ 10 mIU/mL at week 4). Table S2. Comparison of characteristics between responders and non-responders (anti-HBs ≥ 10 mIU/mL) at week 28. [file 12981_2019_225_MOESM1_ESM.docx]

**Table S1: Comparison of characteristics between those who had and did not have anamnestic response (anti-HBs ≥10 mIU/ml at week 4)**

| **Predictive factors** | **No Anamnestic Response (n=38)** | **Anamnestic Response (n=16)** | **P-value** |
| --- | --- | --- | --- |
| Vaccination schedule |  | | 0.551 |
| 3 doses | 20 (52.6%) | 7 (43.8%) |  |
| 4 doses | 18 (47.4%) | 9 (56.3%) |  |
| Age, years (mean ± SD) | 49.4 ± 11.2 | 38.6 ± 11.6 | 0.002 |
| Male | 22 (57.9%) | 10 (62.5%) | 0.753 |
| Risk of HIV acquisition |  | | 0.257 |
| Heterosexual | 30 (79.0%) | 10 (62.5%) |  |
| Homosexual (MSM) | 7 (18.4%) | 5 (31.3%) |  |
| Blood transfusion | 1 (2.6%) | 0 |  |
| IVDU | 0 | 1 (6.3%) |  |
| Time since HIV diagnosis, years (median (IQR)) | 14 (10, 18) | 8.5 (2.5, 14) | 0.006 |
| Duration of cART, years (median (IQR)) | 12 (8, 13) | 7 (2, 11) | 0.002 |
| CD4+ cell count, cells/mm^3^ (median (IQR)) | 609 (432, 829) | 496 (362, 915) | 0.712 |
| Nadir CD4+ cell count, cells/mm^3^ (median (IQR)) | 103 (39, 206) | 198 (134, 291) | 0.029 |
| Nadir CD4+ cell count <200 cells/mm^3^ | 28 (73.7%) | 9 (56.3%) | 0.208 |
| Current cART active against HBV | 38 (100%) | 16 (100%) |  |
| cART regimen |  | | 0.306 |
| NNRTI-based | 34 (89.5%) | 16 (100%) |  |
| PI-based | 4 (10.5%) | 0 |  |

**Abbreviations:** MSM, men who have sex with men; IVDU, intravenous drug use; IQR, interquartile range; SD, standard deviation; cART, combination antiretroviral therapy;

NNRTI, non-nucleoside reverse transcriptase inhibitor; PI: protease inhibitor

**Table S2: Comparison of characteristics between responders and non-responders (anti-HBs ≥10 mIU/ml) at week 28**

| **Predictive factors** | **Non-responder (n=7)** | **Responder (n=47)** | **P-value** |
| --- | --- | --- | --- |
| Vaccination schedule |  | | 1.000 |
| 3 doses | 4 (57.1%) | 23 (48.9%) |  |
| 4-doses | 3 (42.9%) | 24 (51.1%) |  |
| Age, years (mean ± SD) | 49.0 ± 16.8 | 45.8 ± 11.6 | 0.519 |
| Male | 6 (85.7%) | 26 (55.3%) | 0.127 |
| Risk of HIV acquisition |  | | 0.929 |
| Heterosexual | 5 (71.4%) | 35 (74.5%) |  |
| Homosexual (MSM) | 2 (28.6%) | 10 (21.3%) |  |
| Blood transfusion | 0 | 1 (2.1%) |  |
| IVDU | 0 | 1 (2.1%) |  |
| Time since HIV diagnosis, years (mean ± SD) | 10.9 ± 6.8 | 12.0 ± 6.3 | 0.663 |
| Duration of cART, years (mean ± SD) | 9.1 ± 5.8 | 9.8 ± 4.6 | 0.740 |
| CD4+ cell count, cells/mm^3^ (median (IQR)) | 611 (543, 768) | 534 (392, 853) | 0.797 |
| Nadir CD4+ cell count, cells/mm^3^ (median (IQR)) | 52 (13, 228) | 123 (55, 261) | 0.236 |
| Nadir CD4+ cell count <200 cells/mm^3^ | 5 (71.4%) | 32 (68.1%) | 0.859 |
| Current cART active against HBV | 7 (100%) | 47 (100%) |  |
| cART regimen |  | | 1.000 |
| NNRTI-based | 7 (100%) | 43 (91.5%) |  |
| PI-based | 0 | 4 (8.5%) |  |

**Abbreviations:** MSM, men who have sex with men; IVDU, intravenous drug use; IQR, interquartile range; SD, standard deviation; cART, combination antiretroviral therapy;

NNRTI, non-nucleoside reverse transcriptase inhibitor; PI: protease inhibitor
